# Photobiomodulation in the management of oral lesions: a targeted, evidence-based approach

**DOI:** 10.1590/1807-3107bor-2026.vol40suppl1059

**Published:** 2026-08-24

**Authors:** Valesca Sander KOTH, Amanda de Farias GABRIEL, Iohanna DECKMANN, Rogerio Moraes CASTILHO, Felipe NOR, Marcelo Lazzaron LAMERS, José Antonio Poli de FIGUEIREDO, Manoela Domingues MARTINS

**Affiliations:** (a) Universidade Federal of Rio Grande do Sul - UFRGS, School of Dentistry, Department of Oral Pathology, Porto Alegre, RS, Brazil; (b) Universidade Federal of Rio Grande do Sul - UFRGS, Institute of Basic Health Sciences, Department of Morphological Sciences, Porto Alegre, RS, Brazil; (c) University of Michigan School of Dentistry, Department of Periodontics and Oral Medicine, Ann Arbor, MI, United States

**Keywords:** Laser Therapy, Low-Level Light Therapy, Mouth Diseases, Mucositis, Stomatitis

## Abstract

This review synthesizes current literature on laser applications in dentistry, with particular emphasis on the biological mechanisms of photobiomodulation (PBM), dosimetric considerations, tissue interactions, and the clinical management of the most prevalent oral lesions. The oral disorders most frequently investigated for the therapeutic use of PBM were: a) oral pain conditions: trigeminal neuralgia, temporomandibular disorders, and burning mouth syndrome; b) oncology-related conditions: oral mucositis and hyposalivation; c) immune-mediated disorders: recurrent aphthous stomatitis and oral lichen planus; and d) viral infections: herpes labialis. A wide range of laser parameters has shown clinical benefits in the treatment of these disorders. These outcomes are attributed to the anti-inflammatory, analgesic, and tissue repair effects of PBM, which may occur immediately and cumulatively over repeated sessions. PBM therapy is a valuable therapeutic resource in contemporary dentistry and may be successfully applied in a wide range of clinical conditions. Clinical effectiveness of PBM depends on appropriate dosimetry and irradiation parameters according to the biological characteristics of each condition and therapeutic target. Therefore further well-designed randomized clinical trials and protocol standardization are still required to strengthen clinical recommendations and optimize reproducibility.

## Introduction

The use oflasers in dentistry has expanded substantially over recent decades, driven by substantial advances in scientific research and the incorporation of emerging technologies into minimally invasive clinical approaches.^1^ Currently, several laser systems are employed in dental practice, each presenting distinct wavelengths, tissue interactions, and specific clinical indications. Among the most commonly used are diode lasers, helium-neon (HeNe) lasers, neodymiumdoped yttrium aluminum garnet (Nd:YAG) lasers, erbium family lasers, including Er:YAG and Er,Cr:YSGG, and carbon dioxide (CO_2_) lasers. These technologies have been increasingly integrated into multiple dental specialties due to their ability to promote greater precision, reduced intraoperative bleeding, enhanced patient comfort, biomodulation effects, and improved postoperative recovery.^2^ Photobiomodulation (PBM), a term proposed to replace the previously used designation “low-level laser therapy,” is defined as a light-based, non-thermal therapeutic approach that employs laser or light-emitting diode (LED) irradiation to induce biological effects through photophysical and photochemical cellular reactions without causing significant temperature elevation.^3^ PBM acts through the interaction of photons with endogenous intracellular chromophores, leading to modulation of cellular metabolism and activation of signaling pathways associated with anti-inflammatory, analgesic, immunomodulatory, and tissue regenerative effects.^4^, ^5^

From a mechanistic perspective, the biological effects generated by PBM are initiated following photon absorption by endogenous photoacceptors, particularly cytochrome c oxidase (CCO), a key enzyme located within mitochondrial complex IV of the respiratory chain. This interaction promotes photodissociation of inhibitory nitric oxide from CCO, restoration of mitochondrial oxidative phosphorylation, increased adenosine triphosphate (ATP) synthesis, transient production of reactive oxygen species (ROS), and modulation of intracellular calcium signaling. Subsequently, these events activate transcription factors and molecular pathways associated with cellular proliferation, angiogenesis, tissue repair, inflammation modulation, and metabolic regulation.^6^

PBM can be performed using different light sources; however, diode lasers have become the most widely used devices in clinical dentistry due to their portability, lower cost, ease of operation, and broad therapeutic applicability. Diode lasers are semiconductor-based systems composed of compounds containing elements such as gallium, arsenide, aluminum, indium, nitrogen, and phosphorus. The semiconductor materials most commonly employed in dentistry include gallium-aluminum-arsenide (GaAlAs; 780–980 nm), aluminum–gallium–indium–phosphide (AlGaInP; 630–700 nm), indium–gallium–nitride (InGaN; 405–470 nm), and gallium arsenide (GaAs; approximately 904 nm), each responsible for specific wavelength emissions and distinct tissue interaction profiles. Their increasing incorporation into dental practice is largely related to their effectiveness in pain control, inflammatory modulation, wound healing, neural repair, and soft tissue management.^7^

The therapeutic effectiveness of PBM is highly dependent on dosimetry, since the biological response is determined not only by the amount of energy delivered, but also by how this energy is distributed within the target tissue. Therefore, the concept of “dose” in PBM should not be interpreted as an isolated parameter, but rather as the result of a complex interaction among multiple irradiation and tissue-related variables. PBM dosimetry encompasses a constellation of interdependent parameters, including wavelength, power output, irradiance, fluence, exposure time, total energy, beam profile, pulse structure, spot size, treatment interval, delivery mode, and tissue optical properties. These variables collectively determine photon absorption, tissue penetration, light attenuation, and ultimately the magnitude and direction of the biological response.^8^

PBM exhibits a dose-dependent, biphasic response that can be explained, in part, by the Arndt–Schulz law, which represents one of the fundamental biological principles underlying PBM. According to this model, biological systems respond differently depending on the intensity and amount of energy delivered: lower doses generally promote cellular stimulation and metabolic activation, intermediate doses may induce modulatory or inhibitory effects, whereas excessively high doses can suppress cellular function and compromise therapeutic outcomes. Consequently, therapeutic success depends on identifying the optimal therapeutic window capable of eliciting biomodulation without inducing cellular stress or metabolic suppression (Figure).^6^

**Figure:**
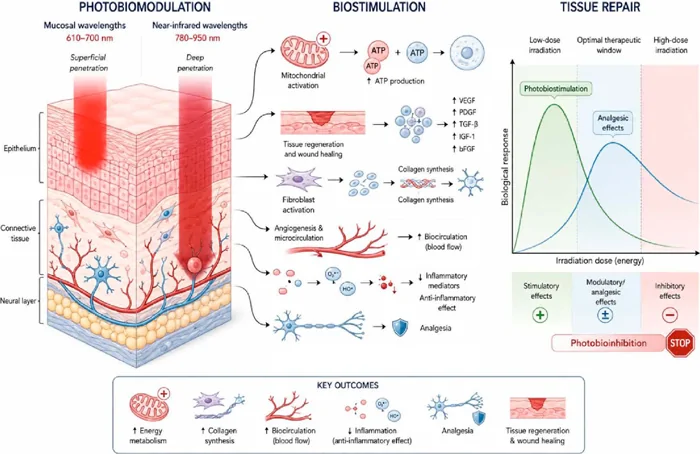
Biological mechanisms and dose-dependent effects of photobiomodulation (PBM) on oral tissues. PBM promotes mitochondrial activation, increased ATP production, collagen synthesis, angiogenesis, anti-inflammatory activity, analgesia, and tissue repair. The figure also illustrates the biphasic dose-response behavior, in which therapeutic effects are achieved within an optimal irradiation window, while excessive energy may induce photobioinhibition.

Therefore, the standardization of PBM protocols remains a major challenge in clinical dentistry and translational research due to the complexity of dosimetric parameters and the variability of laser-tissue interactions. In this context, the present study aims to critically review the current scientific evidence regarding laser applications in dentistry, with particular emphasis on the biological mechanisms of PBM, dosimetric considerations, tissue interactions, and the clinical management of the most prevalent oral lesions.

## Methods

An electronic search was performed to evaluate the landscape of scientific activity, aligning with the study’s objective in MEDLINE/PubMed, Scopus and Cochrane library databases with the terms: LASER OR lasertherapy OR photobiomodulation AND oral lesions OR oral diseases OR mouth OR aphtous ulcer OR mucositis OR lichen planus OR hyposalivation OR herpes labialis OR burning mouth syndrome OR trigeminal neuralgia OR temporomandibular disorders were used as Mesh Terms. Systematic reviews, randomized clinical trials and cohort studies were selected for analysis. Case reports, letter to the editors and reviews were excluded.

## Results

The oral and maxillofacial disorders most frequently investigated for the therapeutic use of PBM can be classified into four main categories: a) oral pain conditions, including, trigeminal neuralgia, temporomandibular disorders and burning mouth syndrome; b) oncology-related conditions, particularly oral mucositis and hyposalivation associated with cancer therapy; c) immune-mediated disorders, such as recurrent aphthous stomatitis and oral lichen planus; and d) viral infections, especially herpes labialis.

The application of laser therapy in these conditions is discussed in detail in the following sections.

### Oral pain conditions

#### Burning Mouth Syndrome

Burning mouth syndrome (BMS) is a chronic neuropathic pain condition characterized by spontaneous, persistent, or recurrent oral burning pain or dysesthesia, frequently associated with xerostomia and occurring in the absence of clinically evident oral lesions. It is currently considered a neuropathic pain disorder. BMS predominantly affects postmenopausal women, and psychological conditions such as anxiety and depression are commonly associated with this disorder.^4^ Conventional treatment strategies include anxiolytics, antidepressants, antiepileptic drugs, topical capsaicin, hormone therapy, and dietary supplementation; however, clinical outcomes are often limited and inconsistent.^9^

In this context, PBM has emerged as a promising therapeutic approach due to its analgesic, anti-inflammatory, and neuromodulatory effects. Two systematic reviews evaluating randomized controlled trials concluded that PBM is a safe and effective therapy for pain reduction in patients with BMS.^4^, ^10^

The chronic nature of BMS usually requires multiple PBM sessions over an extended period. Although immediate pain relief is frequently observed after each irradiation session, effective management generally requires one or two sessions per week for at least four weeks.^4^, ^10^ More significant clinical improvement is commonly observed after the third treatment session, suggesting a cumulative biological effect of PBM over time. Furthermore, pain reduction may persist for up to four months after completion of laser therapy.^4^, ^9^, ^10^

Both red and near-infrared (NIR) wavelengths have demonstrated effectiveness in reducing BMS-related pain.^4^, ^5^ However, NIR wavelengths, particularly 810 nm and 975 nm, appear to be more effective in alleviating numbness and neurosensory alterations. Moreover, a randomized controlled trial comparing different wavelengths demonstrated superior pain reduction with NIR irradiation. While the 660 nm group exhibited a 23.38% reduction in pain scores, the 810 nm and 975 nm groups demonstrated approximately 40% reduction on the visual analog scale (VAS).^5^

Regarding dosimetry, current evidence suggests that, within the therapeutic window, higher energy doses may provide greater analgesic effects compared with lower doses.^4^ Across clinical studies, PBM protocols demonstrated considerable variability, with energy delivery ranging from approximately 0.78 to 12 J per irradiation point and fluence varying between 1 and 200 J/cm^2^ per point, although protocols associated with better clinical outcomes generally applied 4 to 6 J per point and 10 to 50 J/cm^2^ per point.^4^, ^5^ These effects are likely related to modulation of nerve conduction and regulation of neurotransmitter release in nociceptive neurons. Consequently, higher irradiance and energy densities may be more effective for neural inhibition and pain control.^4^, ^10^ Suggested PBM parameters for BMS management include wavelengths ranging from 800 to 830 nm, power output between 60 and 200 mW, exposure times of less than 1 minute per irradiation point, irradiance values of up to 50 mW/cm^2^, energy delivery of approximately 4–6 J per point, and fluence between 10 and 50 J/cm^2^ per point. Common anatomical sites selected for irradiation include the tongue, buccal mucosa, lips, hard palate, soft palate, and alveolar ridge mucosa.^10^

#### Trigeminal neuralgia

Trigeminal neuralgia (TN) is a debilitating neuropathic disorder characterized by unilateral, brief, electric shock-like episodes ofpain with abrupt onset and termination, affecting one or more divisions of the trigeminal nerve. Pain episodes are commonly triggered by innocuous stimuli, such as speaking, chewing, tooth brushing, or light facial touch. Due to its severe intensity and recurrent nature, TN can significantly compromise quality of life, particularly in refractory cases that do not adequately respond to conventional therapies.^11^, ^12^

Current management of TN is primarily based on centrally acting pharmacological agents, especially carbamazepine and gabapentin. Although these medications are considered first-line therapies, their long-term use is frequently associated with adverse effects, including drowsiness, headache, xerostomia, ataxia, cardiovascular alterations, and gastrointestinal disturbances, which may negatively impact patient adherence and overall quality of life.^11^, ^12^

In this context, PBM has emerged as a promising adjunctive therapeutic approach for trigeminal neuralgia. Both low-power lasers and defocused high-power laser approaches have been investigated for pain control and neuromodulation. Clinical studies have demonstrated that irradiation with NIR wavelengths along the affected nerve pathway or directly over trigger points can significantly reduce pain intensity and improve patient comfort.^11^, ^13^

Current evidence suggests that therapeutic outcomes are influenced by PBM dosimetry and treatment frequency. Higher power settings and a greater number of treatment sessions appear to be associated with more pronounced clinical improvement, including substantial pain reduction and decreased dependence on pharmacological therapy.^11^, ^12^. According to a systematic review of randomized controlled trials, the fluence values employed for TN management ranged from 3J/cm^2^ to 12.73 J/cm^2^, while total energy delivery per irradiation point varied between 3 J and 16 J^11^.

Interestingly, PBM combined with carbamazepine demonstrated approximately 53% reduction in pain intensity, compared with only 21% reduction observed with pharmacological therapy alone(11)^11^ These beneficial effects are likely related to PBM-induced modulation ofinflammatory cytokines, improvement of local microcirculation, inhibition of nociceptive transmission, and stimulation of peripheral nerve regeneration^13^.

The absence of significant adverse effects associated with PBM further reinforces its potential as a valuable adjunctive therapy for trigeminal neuralgia. Additionally, considering the complications frequently associated with prolonged pharmacological treatment, PBM may contribute to reducing both medication dosage and therapeutic complexity in affected patients. Nevertheless, despite encouraging results, currently available protocols remain highly heterogeneous, with substantial variability in wavelength selection, dosimetric parameters, irradiation sites, and treatment schedules. Furthermore, long-term randomized clinical trials with larger sample sizes are still limited. Therefore, PBM should presently be considered a complementary therapeutic modality integrated into multidisciplinary management guided by neurologists or pain specialists, rather than a definitive standalone replacement for established medical or surgical treatments.^11^, ^12^.

#### Temporomandibular disorders (TMD)

Temporomandibular disorders (TMDs) represents a group of heterogeneous musculoskeletal disorders that affect the masticatory muscles, the temporomandibular joint (TMJ), and associated structures. As a group, TMDs are characterized by regional pain in the facial and preauricular areas or by limitation or interference in mandibular movement. Frequent clinical findings include hyperalgesia, usually revealed through the application of pressure to the masticatory muscles or TMJs, and TMJ sounds.^14^

PBM therapy has been widely used in the treatment of different inflammatory conditions due to its ability to modulate inflammation, stimulate tissue repair, and reduce pain.^15^ In this context, PBM has been applied as a noninvasive and low-cost therapeutic modality for the management of TMDs. Some clinical studies have reported improvements in maximum mouth opening and significant pain reduction using wavelengths such as 830 nm and 670 nm.^16^, ^17^, ^18^ These findings suggest a potential clinical benefit of PBM in the symptomatic control of pain associated with TMDs, although variability in irradiation parameters and treatment protocols still limits direct comparison among studies. The local analgesic effects of PBM are primarily related to its anti-inflammatory properties, which contribute to the reduction of algogenic substances, stimulation of reflex responses, and release of endogenous opioids such as endorphins involved in pain modulation.^15^ In addition, PBM may improve local micro circulation and blood supply in areas affected by muscle tension.^19^ Furthermore, this therapy has been suggested to influence neuronal membrane stability by acting directly on peripheral nerve endings and prolonging analgesic effects, thereby reducing the transmission of painful stimuli.^15^ Clinical studies evaluating PBM for TMD management have demonstrated positive outcomes regarding pain reduction and functional improvement.^16^, ^20^, ^21^ A systematic review demonstrated that the most effective PBM outcomes for joint disorders are achieved with NIR lasers (820–830 nm) using parameters ranging from 4 to 24 J and 30 to 210 mW/cm^2^ per session.^15^ Furthermore, the recommendations of the World Association for Photobiomodulation Therapy (WALT) for the treatment of TMDs include the use of NIR laser wavelengths between 780 and 820 nm, fluence of 6 J/cm^2^ applied to one or two points every 48 hours over a three to four weeks.^15^

### Oncology related conditions

The use of PBM is well established as an important adjunctive therapy during cancer treatment for the management of oral mucositis and hyposalivation, which are among the most common adverse effects associated with head and neck radiotherapy (HNRT), chemotherapeutic agents, and hematopoietic stem cell transplantation (HSCT). In recent years, PBM has gained substantial scientific recognition due to its ability to reduce pain, modulate inflammation, accelerate tissue repair, and improve patients' quality of life during oncologic therapy.^22^, ^23^

Importantly, international organizations such as the Multinational Association of Supportive Care in Cancer (MASCC) and WALT have published evidence-based clinical guidelines supporting the use of PBM in oncology care. These guidelines recognize PBM as an important component of supportive cancer therapy and currently consider it a standard-of-care interventions for selected indications, particularly for the prevention and management of oral mucositis in patients undergoing HNRT and HSCT. Furthermore, these organizations emphasize the importance of protocol standardization, including wavelength selection, dosimetry, irradiation sites, and treatment schedules, to ensure safe, reproducible, and clinically effective outcomes in oncologic patients.

#### Oral mucositis

Oral mucositis is an inflammatory condition of the oral mucosa characterized by erythema, ulceration, severe pain, and impairment of oral function, frequently compromising speech, swallowing, and nutritional intake. It represents one of the most common and debilitating acute adverse effects associated with HNRT, chemotherapy, and HSCT. In severe cases, oral mucositis may significantly impair patients' quality of life, increase morbidity, predispose to secondary infections, and even lead to interruption or modification of oncologic treatment protocols.^22^, ^23^

PBM has become a well-established supportive therapy for the prevention and management of oral mucositis in oncologic patients due to its ability to accelerate tissue repair, modulate inflammatory responses, reduce oxidative stress, and promote analgesia.^22^, ^23^ In addition to reducing mucosal injury severity, the analgesic effects of PBM contribute to improved swallowing function and maintenance of oral feeding, thereby minimizing nutritional impairment during cancer therapy.^24^

Importantly, the Multinational Association of Supportive Care in Cancer/International Society of Oral Oncology (MASCC/ISOO) recommends intraoral PBM as both a preventive and therapeutic strategy for oral mucositis in patients undergoing HNRT, with or without chemotherapy, as well as HSCT.^25^ Similarly, WALT has reinforced the role of PBM as a standard of care in supportive oncology, emphasizing the importance of evidence-based dosimetry and protocol standardization.

Evidence-based dosimetry and protocol standardization are essential to optimize PBM outcomes in oral mucositis management. Both red wavelengths (630–680 nm) and NIR wavelengths (approximately 780–904 nm) have demonstrated beneficial effects in the prevention and treatment of oral mucositis. PBM protocols are generally initiated at the beginning of oncologic therapy to prevent the onset of mucosal lesions. Red wavelengths have been the most extensively investigated, with favorable outcomes reported using energy delivery ranging from approximately 1 to 4 J per irradiation point and energy densities between 1.5 and 6.3 J/cm^2^ per point, particularly in protocols validated for HSCT and HNRT.^26^, ^27^ NIR wavelengths have also demonstrated favorable clinical effects, typically employing 2 to 6 J per point and fluence values extending up to 10–50 J/cm^2^, depending on tissue depth and clinical indication, suggesting the existence of a relatively broad therapeutic window for PBM in oral mucositis management.^26^, ^27^ Preventive PBM protocols are commonly performed daily until the fifth day after hematopoietic stem cell transplantation or five times per week throughout radiotherapy. Once oral mucositis is established, PBM should be maintained until complete mucosal healing is achieved.

For patients undergoing HSCT, the following PBM parameters have been suggested: wavelength of660 nm, power output of 40 mW, energy delivery of 0.16 J per point, power density of 1 W/cm^2^, energy density of 4 J/cm^2^, and spot size of 0.04 cm^2^.^27^ Using these parameters, PBM demonstrated approximately a 94% greater reduction in oral mucositis severity compared with control groups without laser therapy (p < 0.0002). Furthermore, a meta-analysis of randomized controlled trials reported a significant protective effect of approximately 20% against the development of severe mucositis [confidence interval: 0.02–0.37; heterogeneity: I^2^ = 68% (p = 0.004)].

Although intraoral PBM remains the conventional approach for mucositis prevention and treatment, extraoral PBM protocols have recently emerged as promising alternatives, particularly for patients experiencing pain, limited mouth opening, or trismus resulting from oncologic treatment or surgical sequelae. Extraoral PBM generally employs NIR wavelengths ranging from 800 to 1100 nm and offers practical advantages for both patients and clinicians, including shorter application times and reduced procedural discomfort while maintaining similar clinical effectiveness compared with conventional intraoral PBM protocols.^28^ Evidence supporting the clinical feasibility of extraoral PBM has expanded in recent years. A multicenter, randomized, single-blind clinical trial conducted in patients undergoing treatment for head and neck cancer demonstrated that extraoral PBM was not inferior to intraoral PBM for oral mucositis management, reinforcing its applicability as a clinically viable alternative, particularly for patients with pain, restricted oral access, or poor tolerance to intraoral manipulation.^29^

Current evidence from randomized clinical trials and systematic reviews consistently supports the effectiveness of PBM in both the prevention and management of oral mucositis. Despite the heterogeneity observed among protocols and dosimetric parameters, PBM has been associated with significant reductions in oral mucositis severity and duration. These benefits appear to translate into reduced analgesic demand, particularly lower opioid use, shorter hospital stays, and, consequently, meaningful improvements in patients' quality of life.^25^, ^30^

#### Hyposalivation

Hyposalivation is characterized by a reduction in salivary flow and is frequently accompanied by the subjective sensation of oral dryness known as xerostomia. Although these terms are often used interchangeably, hyposalivation is objectively defined as an unstimulated salivary flow rate lower than 0.1 mL/min or a stimulated salivary flow rate lower than 0.5 mL/min, whereas xerostomia specifically refers to the subjective perception of dry mouth. These conditions may be associated with Sjogren syndrome, systemic medications, metabolic disorders, or sequelae of HNRT, significantly compromising patients' quality of life and oral function^31^.

PBM has emerged as a promising noninvasive therapeutic approach for the management of hyposalivation and xerostomia. Clinical studies have demonstrated that PBM may improve salivary gland function by increasing salivary flow and enhancing saliva quality through modulation of pH, immunoglobulin A (IgA) concentration, and buffering capacity.^31^, ^32^, ^33^

Several studies have investigated different PBM protocols, demonstrating considerable variability in irradiation parameters, wavelengths, treatment frequency, and irradiation sites. Both red and NIR wavelengths have been evaluated either individually or in combination. Some protocols combine red wavelengths for irradiation of minor salivary glands with NIR wavelengths directed toward major salivary glands,^31^, ^32^ whereas other studies have employed only red wavelengths.^34^, ^35^ The number of treatment sessions also varies substantially among studies, which may contribute to inconsistencies in clinical outcomes.

According to Golez et al.,^33^ irradiation of major salivary glands appears to produce superior clinical outcomes compared with exclusively intraoral irradiation, likely due to the substantially greater salivary volume produced by the major glands. Furthermore, the authors emphasized that both excessively low and excessively high cumulative energy doses or energy densities may negatively affect therapeutic outcomes, reinforcing the importance of adequate PBM dosimetry.

A systematic review of randomized controlled trials demonstrated beneficial effects of PBM on radiotherapy-induced hyposalivation. Although the quality of evidence was considered low, the authors observed improvements in salivary flow beginning around the fifteenth fraction of radiotherapy, with even greater benefits at the end of oncologic treatment, suggesting a cumulative dose-dependent effect of PBM on salivary gland function.^31^ Another systematic review including patients with hyposalivation associated with radiotherapy, medications, and diabetes mellitus also reported improvements in salivary flow following PBM therapy.^33^

Preclinical studies suggest that the beneficial effects of PBM on salivary flow are associated with increased ATP availability and modulation of cellular metabolism. In addition, PBM may stimulate epithelial cell proliferation, promote the development of ductal structures, and protein synthesis, reduce inflammatory markers, and improve salivary gland vascularization and secretory activity. Collectively, these findings suggest that PBM may modulate the functional capacity of glandular parenchyma and stimulate salivary production during treatment.^31^, ^32^, ^33^, ^34^, ^35^, ^36^ However, it is important to emphasize that residual glandular viability is essential for achieving favorable therapeutic outcomes. PBM is unlikely to restore salivary flow in cases of irreversible glandular damage, such as advanced acinar atrophy or extensive fibrosis, where residual functional tissue is absent.^33^ This limitation may explain why some studies failed to demonstrate increased salivary flow in patients with Sjogren syndrome following PBM therapy.^37^

Current literature demonstrates conflicting findings regarding the effects of PBM on xerostomia. While several studies have reported improvements in xerostomia symptoms and quality oflife,^32^, ^33^, ^37^, ^38^ other investigations failed to observe significant benefits.^31^, ^39^ Since xerostomia is a subjective symptom associated with multiple etiologies, its evaluation remains inherently complex and susceptible to bias. Consequently, adjunctive assessment tools such as visual analogue scales and quality-of-life questionnaires are frequently employed in clinical studies. Despite the heterogeneity of available evidence, PBM has generally been associated with improved quality oflife in patients affected by xerostomia. Nevertheless, the substantial variability in laser parameters and treatment protocols among studies likely contributes to the current lack of consensus regarding the effectiveness of PBM for xerostomia management.

Regarding PBM dosimetry for xerostomia and hyposalivation, considerable heterogeneity has been reported across studies, particularly with respect to wavelength, energy delivery, irradiation sites, and cumulative dose. Most clinical protocols employ red wavelengths (630–685 nm), NIR wavelengths (780–904 nm), or combinations of both, with irradiation directed either intraorally toward minor salivary glands or extraorally toward major salivary glands. Energy delivery has generally ranged from approximately 0.5 to 6 J per irradiation point, with fluence values varying between 4 and 50 J/cm^2^ per point, depending on gland depth and treatment objectives.^31^, ^33^ Studies employing irradiation of the major salivary glands, particularly using NIR wavelengths to optimize tissue penetration, have frequently reported superior clinical outcomes compared with exclusively intraoral protocols^33^. Importantly, evidence suggests the existence of a dose-dependent therapeutic window, as both insufficient and excessively high cumulative energy doses may negatively affect treatment response, reinforcing the importance of individualized and biologically appropriate dosimetry.^32^, ^33^

### Immune-mediated disorders

#### Recurrent aphthous stomatitis

Recurrent aphthous stomatitis (RAS) represents a common inflammatory condition characterized by the recurrent development of painful ulcerative lesions affecting the oral mucosa. RAS is an immune-mediated disorder in which round well-defined ulcers surrounded by erythematous halo develop in non-keratinized oral mucosal until spontaneous recovery.^40^ Pain is the major symptom which causes significant discomfort, impairing essential oral functions such as eating, swallowing, and speech.^7^, ^40^, ^41^ The disorder can be classified according to the pattern of the lesions as minor, major or herpetiform. Minor RAS is the most common presentation of this disorder and is characterized by the development of a solitary or a few superficial ulcers smaller than 1cm that last for 5 to 21 days. The major RAS shows deeper ulcers larger than 1cm that can last for weeks or months and the herpetiform RAS presents multiple small ulcers (1- 2mm).^40^ RAS is more frequently observed in children and young adults, although studies have included patients ranging from 5 to 60 years of age.^7^

Conventional management of RAS is primarily based on topical or systemic corticosteroids. In addition, other therapeutic approaches, including immunosuppressive agents, nutritional supplementation, antiseptics, anti-inflammatory drugs, and topical antibiotics, have also been employed. Nevertheless, currently available therapies remain predominantly symptomatic rather than curative.^41^

In recent years, different laser modalities and irradiation parameters have been extensively investigated for RAS management, particularly aiming to promote pain relief and accelerate ulcer healing, frequently as an alternative to corticosteroid therapy. Current evidence includes randomized clinical trials and several systematic reviews demonstrating that both low-power and high-power defocused laser therapies provide superior outcomes in terms of pain reduction and healing time when compared with conventional treatments^42^. Notably, even a single laser irradiation session may significantly accelerate lesion healing and improve patient comfort.^7^

Pain represents the most relevant clinical symptom associated with RAS, and PBM has demonstrated the ability to induce immediate analgesic effects. Furthermore, analyses using the VAS consistently demonstrate lower pain scores in patients treated wtih PBM compared with those receiving conventional therapies during follow-up periods.^42^ The rationale for laser application in RAS management is associated with stimulation of re-epithelialization, enhanced vascularization, increased collagen synthesis, and neuromodulatory effects that contribute to significant analgesia.^41^

Regarding irradiation parameters, favorable clinical outcomes have been reported using wavelengths ranging from 610 to 980 nm, with both red and NIR light demonstrating therapeutic benefits. However, NIR wavelengths appear to promote faster healing responses compared with red wavelengths, particularly regarding ulcer resolution time.^7^, ^42^ Across randomized clinical trials, PBM protocols demonstrated substantial variability, with energy delivery generally ranging from approximately 0.5 to 6 J per irradiation point and fluence values varying between 1 and 75 J/cm^2^, although the majority of successful protocols concentrated between 2 and 4 J per point and approximately 4 to 10 J/cm^2^.^41^, ^42^ Importantly, comparative analyses between energy densities below and above 10 J/cm^2^ did not reveal significant differences in healing outcomes, suggesting that excessively high fluences may not be necessary to achieve therapeutic effectiveness in recurrent aphthous stomatitis.^7^, ^42^ Irradiation protocols generally recommend inclusion of both the aphthous lesion and the adjacent mucosa, extending approximately 0.5–1 cm beyond ulcer margins, within the treatment field. PBM with diode lasers is commonly performed using wavelengths between 610 and 980 nm and irradiation times ranging from 30 to 60 seconds per session; in many cases, a single treatment session may induce clinically significant analgesia and accelerate wound healing.^42^ High-power CO_2_ lasers operating at a wavelength of 10,600 nm have also demonstrated favorable clinical results in RAS treatment. However, due to their significantly higher power output and thermal interaction with tissues, irradiation times should be restricted to approximately 5 to 10 seconds to avoid excessive thermal damage.^42^

Current literature supports the use of PBM, for the management of recurrent aphthous ulcers. The analgesic effects, reduction in lesion size, and acceleration of healing observed with PBM demonstrate outcomes comparable or even superior to those achieved with topical corticosteroids. Nevertheless, treatment protocols remain heterogeneous, and factors such as device availability, treatment cost, and operator expertise should still be considered when incorporating laser therapy into routine clinical practice.

#### Oral lichen planus

Oral lichen planus (OLP) is a chronic immune-mediated mucocutaneous disorder that commonly presents as white reticular lesions, erythematous areas, and painful erosive ulcers affecting the oral mucosa. The disease is characterized by a relapsing-remitting clinical course and carries a small but clinically relevant risk of malignant transformation.^43^, ^44^ Depending on the clinical presentation, OLP can be asymptomatic or symptomatic. The reticular OLP is characterized by Wickham striae and is asymptomatic while the erosive OLP is characterized by loss of epithelium integrity and atrophic OLP shows erythema of the oral mucosa. The atrophic and erosive OLP can cause significant pain and the same patient can show all the patterns combined.^43^, ^45^

Current literature includes several randomized clinical trials and systematic reviews investigating the effects oflaser therapy on OLP, with increasingly promising clinical outcomes. The available evidence on laser-based therapies for OLP has been derived predominantly from studies involving symptomatic forms of OLP, particularly erosive and atrophic subtypes.

Different high-power laser systems have been investigated for the treatment of OLP, including Nd:YAG, CO_2_, Er:YAG, and Er,Cr:YSGG lasers. Nevertheless, most available studies report favorable outcomes using diode laser PBM. A meta-analysis of clinical trials concluded that PBM demonstrates effectiveness comparable to pharmacological therapies in the management of OLP, while presenting fewer adverse effects. Although no statistically significant difference in pain reduction was observed between PBM and topical corticosteroids overall, subgroup analyses based on treatment frequency demonstrated that protocols involving more than 10 PBM sessions resulted in significantly greater symptom improvement, whereas protocols with fewer than 10 sessions achieved outcomes similar to those of corticosteroid therapy.^46^ These findings suggest a dose-dependent response associated with PBM treatment frequency.

PBM has demonstrated clinically meaningful analgesic effects in symptomatic OLP patients, with significant reductions in VAS pain scores reported in several studies.^46^, ^47^ The beneficial effects of PBM are mainly attributed to modulation of inflammatory pathways, reduction of oxidative stress, stimulation of epithelial repair, and improvement of local microcirculation.

Regarding irradiation parameters, considerable heterogeneity has been observed among PBM protocols for oral lichen planus. Favorable clinical outcomes have been reported using wavelengths ranging from approximately 630 to 980 nm, predominantly employing diode lasers in the red and NIR spectrum. Across randomized clinical trials, energy delivery generally ranged from approximately 0.5 to 6 J per irradiation point, while fluence values varied between 1 and 150 J/cm^2^, depending on lesion severity, irradiation technique, and treatment objectives.^46^, ^47^ Nevertheless, protocols associated with more consistent symptom improvement commonly employed approximately 1-4 J per point and 4–10 J/cm^2^, administered over multiple treatment sessions, often exceeding 10 sessions, particularly in erosive or symptomatic OLP^46^. This cumulative treatment effect may partially explain why PBM protocols involving higher session frequency demonstrated superior clinical outcomes compared with topical corticosteroids in subgroup analyses.

In addition, high-power laser therapy in a defocused mode also appears to provide superior outcomes compared with topical corticosteroids, particularly in erosive forms of OLP. Studies have demonstrated greater pain reduction, faster lesion resolution, and lower recurrence rates, suggesting improved long-term disease control^44^. These effects are likely associated with the selective ablation of altered epithelium, reduction of inflammatory infiltrate, and stimulation of tissue remodeling and functional healing.^43^

Overall, PBM may be considered a valuable alternative to corticosteroid therapy for the management of oral lichen planus, particularly in patients who experience adverse effects or contraindications related to prolonged pharmacological treatment. Furthermore, high-power laser therapy has demonstrated promising results, especially in erosive and refractory cases of OLP, reinforcing the growing role of laser-assisted therapies in contemporary oral medicine.^44^, ^46^

### Viral infections

#### Herpes labialis

Recurrent herpes labialis is one of the most common viral infections of the orofacial region. It is caused by reactivation of the herpes simplex virus type 1 (HSV-1), which remains dormant in sensory ganglia after the primary infection. This condition is characterized by the formation of erythema, edema, vesicles, and ulcers on the lips, causing pain, itching, and negatively affecting the patient’s quality oflife. Reactivation is commonly associated with triggers such as stress, immunosuppression, trauma, ultraviolet exposure, or systemic illness, and lesions often recur at or near the original site.^48^, ^49^

The most common treatment for recurrent herpes labialis is systemic or topic use of antiviral compounds, such as acyclovir or valacyclovir. However, repeated or prolonged antivirus use may contribute to drug resistance, particularly in immunocompromised patients.^49^ Accordingly, both PBM and high-power laser therapy have been investigated for the management of herpes labialis with promising results.

Because laser parameters may vary according to lesion stage, recurrent herpes labialis should be classified clinically as prodromal, vesicular, ulcerative, crusting, or healing before treatment. The presence of vesicles indicates risk of transmission of infection, as each vesicle contains infectious viral particles.^50^ In this stage of the infection, the main objective of laser therapy is to decontaminate the area. This may be achieved using antimicrobial photodynamic therapy (aPDT) or with high -power laser, such as Er,Cr:YSGG laser or defocused high-power diode laser.^48^, ^51^ The use of Er,Cr:YSGG on the acute phase of herpes has the advantage of disrupting and draining the vesicles with concomitant air-water spray that can reduce pain during irradiation. However, other high-intensity lasers, such as diode lasers, can be used provided that the operator carefully monitors tissue temperature and uses cooling measures, such as saline-soaked gauze, when necessary.^51^

Regarding irradiation parameters, recurrent herpes labialis management requires stage-specific laser protocols. During the vesicular phase, the primary therapeutic objective is lesion decontamination and viral load reduction. In this context, aPDT is commonly performed using red light (660 nm) associated with methylene blue or toluidine blue photosensitizers at concentrations ranging from 0.005% to 0.01%, usually following a pre-irradiation period of approximately 5 minutes. Reported aPDT protocols generally employ power outputs between 40 and 100 mW, energy delivery ranging from approximately 0.4 to 6 J per irradiation point, and fluence values between 100 and 142.85 J/cm^2^, typically requiring only a single treatment session to achieve immediate pain relief and accelerate lesion healing compared with conventional antiviral therapy^50^. During the crusting and healing phases, PBM aims to stimulate epithelial repair, collagen organization, and analgesia. Therapeutic protocols commonly employ red and NIR wavelengths (632–870 nm) with approximately 1–4 J per irradiation point and fluence values ranging from 2.04 to 48 J/cm^2^, applied daily or every 48 hours until lesion remission.^47^ Importantly, preventive PBM has also demonstrated promising outcomes for recurrent herpes labialis. A randomized controlled study by Zanella et al. 52 employed 808 nm NIR PBM, 100 mW output power, and preventive doses of 1 or 2 J per irradiation point, demonstrating significant reductions in recurrence frequency and lesion severity over a 2-year follow-up period. Interestingly, the 1 J/point protocol demonstrated superior preventive performance compared with 2 J/point, suggesting the existence of an optimal therapeutic window rather than a linear dose-response relationship in herpes recurrence prevention.

The sequential use of aPDT during the vesicular phase followed by PBM during the crusting and healing stages may represent a promising therapeutic strategy for recurrent herpes labialis, as it combines viral decontamination, immediate pain relief, accelerated tissue repair, and improved functional healing.^50^ Overall, laser-based therapies have demonstrated the ability to reduce pain intensity, shorten lesion duration, accelerate re-epithelialization, and improve patient comfort when compared with conventional antiviral approaches.^47^, ^48^ Furthermore, growing evidence suggests that preventive PBM protocols, particularly using NIR wavelengths, may reduce the frequency and severity of recurrent outbreaks and prolong asymptomatic intervals, supporting the role of PBM not only as a therapeutic modality for active lesions but also as a preventive strategy for recurrent herpes labialis management.^52^

## Discussion

Over the last decades, appropriate use of laser therapy has shown positive outcomes on the treatment of different oral lesions without significant adverse effects.^5^ PBM is a safe, well-tolerated therapy that can promote anti-inflammatory, analgesic, and tissue repair effects.^4^ Although the analgesic effects of PBM are immediate, an increased number of PBM sessions is associated with cumulative effects on pain relief with long-lasting effects that may persist after treatment completion.^4^, ^42^

Several laser wavelengths are currently available, and their selection is based on their interaction with tissue components including chromophores, their penetration depth within tissues, and the energy delivered. These factors may determine the therapeutic effect of lasers on each specific condition. The red light is primarily absorbed by superficial chromophores such as hemoglobin and melanin. Because red light has relatively limited tissue penetration, its energy is concentrated near the tissue surface, allowing for good interaction with vascular and pigmented areas, promoting mucosal regeneration and localized pain relief. On the other hand, NIR light generally penetrates more deeply than red light because of lower absorption by superficial chromophores; depending on the wavelength, it may interact with mitochondrial chromophores and tissue water, thereby influencing blood flow, oxygenation, and cellular metabolism. These effects may potentially reduce chronic pain and inflammation.^3^ The analgesic effects of PBM occur through mechanisms such as decreased nerve conduction velocity, increased endorphin production, neural modulation, and reduced production of inflammatory mediators.^32^ Interestingly, it has been pointed out that laser therapy may be more costly when compared to medications. However, the capacity of laser therapy to promote a late effect in pain relief that could last for several months may actually be more financially advantageous when compared to the cost of long-term drug therapy.^5^

Even though considered as a safe therapy by literature, PBM should never be applied indiscriminately. That means that PBM must be preceded by a comprehensive anamnesis and proper clinical evaluation that allows for the establishment of a diagnosis. This step is vital when managing oral mucosal lesions, especially when malignant neoplasms are part of the differential diagnosis. A rigorous upfront assessment is the only way to ensure precise application of photobiomodulation therapy.^8^

A wide range of laser parameters has been associated with positive outcomes across different oral lesions (Table). This represents the so-called therapeutic window, showing that the use of laser can frequently bring clinical benefits without significant adverse effects. The challenge now is to design studies capable of identifying optimal parameters for each condition, maximizing therapeutic efficacy and reduce chairside time. Current literature shows that PBM has positive results on oral disorders, with well-established indications for the management of oral mucositis and recurrent aphthous stomatitis. However, there are still conflicting results for some disorders, such as oral lichen planus, xerostomia, trigeminal neuralgia, and burning mouth syndrome, in which laser appears to be a promising adjuvant option even though there is a significant lack in standardized protocols. In this way, since there is a dose-dependent response to laser, it is imperative to develop new randomized controlled trials to investigate different laser parameters as well as the number of sessions and intervals between sessions for these disorders.

**Table:** Evidence-based photobiomodulation dosimetry according to oral clinical condition.

Clinical condition	Wavelength (nm)	Power output (mW)	Energy per point (J)	Fluence (J/cm^2^)	Treatment frequency	Key consideration
Burning Mouth Syndrome (BMS)	660–975 (preferably 800–975)	60–200	0.78–12 (optimal: 4–6)	1–200 (optimal: 10–50)	1–2 sessions/week for ≤4 weeks	NIR wavelengths show superior pain reduction and improvement of neurosensory symptoms
Trigeminal neuralgia	NIR	Variable	3–16	3–12.73	Multiple sessions	Higher number of sessions shows superior pain reduction
TMD	780–830	Variable	4–24	~6	Every 48 h for 3–4 weeks	NIR lasers demonstrated superior outcomes for pain and mandibular function
Oral mucositis	630–680 / 780–904	~40	0.16–6	1.5–50	Daily during HNRT / HSCT/chemotherapy	Preventive and therapeutic effects supported by MASCC/ISOO and WALT guidelines
Hyposalivation	630–685 / 780–904	Variable	0.5–6	4–50	Variable	Combined red + NIR protocols and major salivary gland irradiation appear to be more effective
RAS	610–980	Variable	0.5–6 (most common: 2–4)	1–75 (most common: 4–10)	Often single session	Similar outcomes observed with fluences below and above 10 J/cm^2^
OLP	630–980	Variable	0.5–6 (most common: 1–4)	1–150 (most common: 4–10)	Usually >10 sessions	Greater cumulative exposure associated with significant symptom improvement
Herpes Labialis – aPDT (vesicular phase)	660	40–100	0.4–6	100–142.85	Usually single session	Red laser associated with 0.005–0.01% methylene blue or toluidine blue for viral decontamination
Herpes Labialis (crusting/healing)	632–870	15–100	1–4	2.04–48	Daily or every 48 h	Red and NIR PBM accelerate epithelial repair and pain control
Herpes Labialis – Preventive	808	100	1–2	~33.3	Preventive protocol	1 J/point demonstrated superior outcomes compared with 2 J/point in recurrence prevention

NIR: near-infrared; TMD: temporal mandibular disorders; HNRT/HSCT: head and neck radiotherapy/hematopoietic stem cell transplantation; RAS: recurrent aphtous stomatitis; OLP: oral lichen planus.Values represent ranges reported across randomized clinical trials and systematic reviews. Preferred dosimetric ranges reflect protocols associated with more consistent clinical outcomes.

## Conclusion

PBM therapy represents a valuable therapeutic resource in contemporary dentistry and may be successfully applied in a wide range of clinical conditions. Nevertheless, its clinical effectiveness is highly dependent on appropriate dosimetry and careful selection of irradiation parameters according to the biological characteristics of each condition and therapeutic target. Current evidence indicates that PBM should not be applied empirically, but rather accordingly to scientific evidence and validated protocols. Despite encouraging outcomes, further well-designed randomized clinical trials and standardized protocols are required to strengthen clinical recommendations and optimize reproducibility. Continued advancement of technical and scientific knowledge in PBM will be essential for its safe, predictable, and evidence-based incorporation into routine dental care.

## Data Availability

The authors declare that all data generated or analyzed during this study are included in this published article.
